# Supplementary material for: A stable, distributed code for cue value in mouse cortex during reward learning
Source: eLife. 2023 Jun 30;12:RP84604. doi: 10.7554/eLife.84604 (PMC10328514; doi:10.7554/eLife.84604)
Supplement: Supplementary file 2. — Top: Bonferroni-corrected p-values from pairwise comparisons between the decoding accuracy of each group of neurons at each time point with their performance at baseline and with the other neuron groups at that time point. Middle, Bottom: Bonferroni-corrected p-values for pairwise comparisons of bootstrapped distributions (1000 samples) of decoding performance using increasing numbers of neurons in each group. [file elife-84604-supp2.docx]

| **Cue decoding performance, Bonferroni corrected p-values for group comparisons (Figure 3G)** | | | | | | | | | | | | |
| --- | --- | --- | --- | --- | --- | --- | --- | --- | --- | --- | --- | --- |
| **Time from cue ->** | -0.25 | 0 | 0.25 | 0.5 | 0.75 | 1 | 1.25 | 1.5 | 1.75 | 2 | 2.25 | 2.5 |
| Value>baseline |  | 1 | 1 | 1 | 2.61E-19 | 5.39E-71 | 3.97E-99 | 1.08E-90 | 5.01E-96 | 3.70E-94 | 4.85E-101 | 1.00E-97 |
| Value like > baseline |  | 1 | 1 | 1 | 3.96E-25 | 1.86E-87 | 1.14E-102 | 8.10E-107 | 1.61E-99 | 1.33E-107 | 2.22E-109 | 7.68E-103 |
| Untuned > baseline |  | 1 | 1 | 1 | 2.61E-19 | 5.39E-71 | 3.97E-99 | 1.08E-90 | 5.01E-96 | 3.70E-94 | 4.85E-101 | 1.00E-97 |
| Value>value like | 1 | 1 | 1 | 1 | 1 | 2.70E-05 | 2.53E-13 | 1.52E-08 | 8.42E-13 | 8.92E-10 | 2.75E-12 | 1.02E-11 |
| Value>untuned | 1 | 1 | 1 | 1 | 1.43E-06 | 1.13E-34 | 2.55E-44 | 1.34E-39 | 1.16E-46 | 1.62E-48 | 3.15E-43 | 2.00E-29 |
| Value like>untuned | 1 | 1 | 1 | 1 | 0.00107 | 6.16E-20 | 4.00E-17 | 3.00E-19 | 1.53E-19 | 3.94E-25 | 1.89E-17 | 5.53E-08 |
| **Cue decoding (population), Bonferroni corrected p-values for group comparisons (Figure 3H)** | | | | | | | | | | | | |
| **Pseudoensemble ->** | 1 | 5 | 10 | 25 | 50 | 75 | 100 | 200 |  |  |  |  |
| Value>chance | 0.012 | 0.001 | 0.001 | 0.001 | 0.001 | 0.001 | 0.001 | 0.001 |  |  |  |  |
| Value like>chance | 0.019 | 0.001 | 0.001 | 0.001 | 0.001 | 0.001 | 0.001 | 0.001 |  |  |  |  |
| Untuned>chance | 0.102 | 0.001 | 0.001 | 0.001 | 0.001 | 0.001 | 0.001 | 0.001 |  |  |  |  |
| Value>value like | 1 | 1 | 1 | 1 | 1 | 1 | 1 | 1 |  |  |  |  |
| Value>untuned | 1 | 0.44 | 0.32 | 0.1428 | 0.123 | 0.1662 | 0.2028 | 0.726 |  |  |  |  |
| Value like>untuned | 1 | 0.86 | 0.62 | 0.1896 | 0.05712 | 0.02832 | 0.021 | 0.05928 |  |  |  |  |
| Value<value like | 1 | 1 | 1 | 1 | 1 | 1 | 0.918 | 0.828 |  |  |  |  |
| Value<untuned | 1 | 1 | 1 | 1 | 1 | 1 | 1 | 1 |  |  |  |  |
| Value like<untuned | 1 | 1 | 1 | 1 | 1 | 1 | 1 | 1 |  |  |  |  |
| **Value decoding (population), Bonferroni corrected p-values for group comparisons (Figure 3I)** | | | | | | | | | | | | |
| **Pseudoensemble ->** | 1 | 5 | 10 | 25 | 50 | 75 | 100 | 200 |  |  |  |  |
| Value>chance | 0.03 | 0.001 | 0.001 | 0.001 | 0.001 | 0.001 | 0.001 | 0.001 |  |  |  |  |
| Value like>chance | 0.031 | 0.001 | 0.001 | 0.001 | 0.001 | 0.001 | 0.001 | 0.001 |  |  |  |  |
| Untuned>chance | 0.126 | 0.001 | 0.001 | 0.001 | 0.001 | 0.001 | 0.001 | 0.001 |  |  |  |  |
| Value>value like | 1 | 0.72 | 0.27 | 0.052 | 0.0162 | 0.0108 | 0.00642 | 7.02E-04 |  |  |  |  |
| Value>untuned | 1 | 0.28 | 0.15 | 0.0068 | 6.00E-06 | 1.20E-05 | 6.00E-06 | 6.00E-06 |  |  |  |  |
| Value like>untuned | 1 | 1 | 1 | 1 | 1 | 1 | 0.93 | 1 |  |  |  |  |
| Value<value like | 1 | 1 | 1 | 1 | 1 | 1 | 1 | 1 |  |  |  |  |
| Value<untuned | 1 | 1 | 1 | 1 | 1 | 1 | 1 | 1 |  |  |  |  |
| Value like<untuned | 1 | 1 | 1 | 1 | 1 | 1 | 1 | 1 |  |  |  |  |
