# Supplementary material for: A stable, distributed code for cue value in mouse cortex during reward learning
Source: eLife. 2023 Jun 30;12:RP84604. doi: 10.7554/eLife.84604 (PMC10328514; doi:10.7554/eLife.84604)
Supplement: Supplementary file 3. — Top, Middle: Bonferroni-corrected p-values from region contrasts in generalized linear mixed-effects model. Bottom: Bonferroni-corrected p-values for pairwise comparisons of bootstrapped distributions (1000 samples) of decoding performance using value cells from each region. [file elife-84604-supp3.docx]

| **Value cells, Bonferroni corrected p-values for pairwise region comparisons (Figure 4A)** | | | | | | | | | |
| --- | --- | --- | --- | --- | --- | --- | --- | --- | --- |
|  | ALM | ACA | FRP | PL | ILA | ORB | DP | TTd | AON |
| ALM |  | 0.0365 | 1 | 0.22 | 1 | 0.0282 | 1 | 1 | 1 |
| ACA | 0.0365 |  | 1 | 1 | 1 | 1 | 0.64 | 0.14 | 0.30 |
| FRP | 1 | 1 |  | 1 | 1 | 1 | 1 | 1 | 1 |
| PL | 0.22 | 1 | 1 |  | 1 | 1 | 1 | 0.90 | 1 |
| ILA | 1 | 1 | 1 | 1 |  | 1 | 1 | 1 | 1 |
| ORB | 0.0282 | 1 | 1 | 1 | 1 |  | 1 | 0.50 | 0.74 |
| DP | 1 | 0.64 | 1 | 1 | 1 | 1 |  | 1 | 1 |
| TTd | 1 | 0.14 | 1 | 0.90 | 1 | 0.50 | 1 |  | 1 |
| AON | 1 | 0.30 | 1 | 1 | 1 | 0.74 | 1 | 1 |  |
|  | Motor | PFC | Olf. |  |  |  |  |  |  |
| Motor |  | 0.0062 | 1 |  |  |  |  |  |  |
| PFC | 0.0062 |  | 0.0002 |  |  |  |  |  |  |
| Olfactory | 1 | 0.0002 |  |  |  |  |  |  |  |
| **Value-like cells, Bonferroni corrected p-values for pairwise region comparisons (Figure 4B)** | | | | | | | | | |
|  | ALM | ACA | FRP | PL | ILA | ORB | DP | TTd | AON |
| ALM |  | 1 | 1 | 0.26 | 0.36 | 1.10E-05 | 0.08 | 0.0002 | 0.0407 |
| ACA | 1 |  | 1 | 1 | 1 | 1 | 1 | 1 | 1 |
| FRP | 1 | 1 |  | 1 | 1 | 0.0443 | 1 | 0.0494 | 0.67 |
| PL | 0.26 | 1 | 1 |  | 1 | 0.36 | 1 | 0.22 | 1 |
| ILA | 0.36 | 1 | 1 | 1 |  | 1 | 1 | 0.89 | 1 |
| ORB | 1.10E-05 | 1 | 0.0443 | 0.36 | 1 |  | 1 | 1 | 1 |
| DP | 0.08 | 1 | 1 | 1 | 1 | 1 |  | 1 | 1 |
| TTd | 0.0002 | 1 | 0.0494 | 0.22 | 0.89 | 1 | 1 |  | 1 |
| AON | 0.04 | 1 | 0.67 | 1 | 1 | 1 | 1 | 1 |  |
|  | Motor | PFC | Olf. |  |  |  |  |  |  |
| Motor |  | 2.34E-05 | 1.17E-07 |  |  |  |  |  |  |
| PFC | 2.34E-05 |  | 0.0443 |  |  |  |  |  |  |
| Olfactory | 1.17E-07 | 0.0443 |  |  |  |  |  |  |  |
| **Population decoding of value, region comparisons (bootstrap, Bonferroni corrected p-values) (row > column) (Figure 4E)** | | | | | | | | | |
|  | ALM | ACA | FRP | PL | ILA | ORB | DP | TTd | AON |
| ALM |  | 1 | 1 | 1 | 1 | 1 | 1 | 1 | 1 |
| ACA | 1 |  | 1 | 1 | 1 | 1 | 1 | 1 | 1 |
| FRP | 1 | 1 |  | 1 | 1 | 1 | 1 | 1 | 1 |
| PL | 1 | 1 | 1 |  | 1 | 1 | 1 | 1 | 1 |
| ILA | 1 | 1 | 1 | 1 |  | 1 | 1 | 1 | 1 |
| ORB | 1 | 1 | 1 | 1 | 1 |  | 1 | 1 | 1 |
| DP | 1 | 1 | 1 | 1 | 1 | 1 |  | 1 | 1 |
| TTd | 1 | 1 | 1 | 1 | 1 | 1 | 1 |  | 1 |
| AON | 1 | 1 | 1 | 1 | 1 | 1 | 1 | 0.680148 |  |
|  | Motor | PFC | Olf. |  |  |  |  |  |  |
| Motor |  | 1 | 1 |  |  |  |  |  |  |
| PFC | 0.23 |  | 0.74 |  |  |  |  |  |  |
| Olfactory | 0.70 | 1 |  |  |  |  |  |  |  |
