# Supplementary material for: A stable, distributed code for cue value in mouse cortex during reward learning
Source: eLife. 2023 Jun 30;12:RP84604. doi: 10.7554/eLife.84604 (PMC10328514; doi:10.7554/eLife.84604)
Supplement: Supplementary file 4. — Top: Bonferroni-corrected p-values for pairwise comparisons of bootstrapped distributions (5000 samples) of the slope of population activity of each group of neurons across CS50 trials of increasing value. Bottom: Bonferroni-corrected p-values from region contrasts in generalized linear mixed-effects model. [file elife-84604-supp4.docx]

| **Slope, Bonferroni corrected p-values (row > column) (Figure 5H)** | | | | | | | | | |
| --- | --- | --- | --- | --- | --- | --- | --- | --- | --- |
|  | history | value | value-like | untuned | licks |  |  |  |  |
| history |  | 2.00E-07 | 2.00E-07 | 2.00E-07 | 0.12 |  |  |  |  |
| value | 1 |  | 0.01 | 1 | 1 |  |  |  |  |
| value-like | 1 | 1 |  | 1 | 1 |  |  |  |  |
| untuned | 1 | 1 | 0.066 |  | 1 |  |  |  |  |
| licks | 1 | 2.00E-07 | 2.00E-07 | 0.0057 |  |  |  |  |  |
| **History cells, Bonferroni corrected p-values for pairwise region comparisons (Figure 5I)** | | | | | | | | | |
|  | ALM | ACA | FRP | PL | ILA | ORB | DP | TTd | AON |
| ALM |  | 1 | 1 | 1 | 1 | 1 | 1 | 1 | 1 |
| ACA | 1 |  | 1 | 1 | 1 | 1 | 0.66 | 1 | 1 |
| FRP | 1 | 1 |  | 1 | 1 | 1 | 1 | 1 | 1 |
| PL | 1 | 1 | 1 |  | 1 | 1 | 1 | 1 | 1 |
| ILA | 1 | 1 | 1 | 1 |  | 1 | 1 | 1 | 1 |
| ORB | 1 | 1 | 1 | 1 | 1 |  | 1 | 1 | 1 |
| DP | 1 | 0.66 | 1 | 1 | 1 | 1 |  | 1 | 1 |
| TTd | 1 | 1 | 1 | 1 | 1 | 1 | 1 |  | 1 |
| AON | 1 | 1 | 1 | 1 | 1 | 1 | 1 | 1 |  |
|  | Motor | PFC | Olf. |  |  |  |  |  |  |
| Motor |  | 0.0048 | 0.85 |  |  |  |  |  |  |
| PFC | 0.0048 |  | 0.0016 |  |  |  |  |  |  |
| Olfactory | 0.85 | 0.0016 |  |  |  |  |  |  |  |
