# Supplementary material for: A stable, distributed code for cue value in mouse cortex during reward learning
Source: eLife. 2023 Jun 30;12:RP84604. doi: 10.7554/eLife.84604 (PMC10328514; doi:10.7554/eLife.84604)
Supplement: Supplementary file 5. — Bonferroni-corrected p-values from region contrast in generalized linear mixed-effects model. [file elife-84604-supp5.docx]

| **Value cells (prop cue), Bonferroni corrected p-values for pairwise region comparisons (Figure 4-figure supplement 2, left)** | | | | | | | | | |
| --- | --- | --- | --- | --- | --- | --- | --- | --- | --- |
|  | ALM | ACA | FRP | PL | ILA | ORB | DP | TTd | AON |
| ALM |  | 1 | 1 | 1 | 1 | 1 | 0.17 | 0.0187 | 0.0549 |
| ACA | 1 |  | 1 | 1 | 0.17 | 0.20 | 0.0094 | 0.0010 | 0.0043 |
| FRP | 1 | 1 |  | 1 | 1 | 1 | 1 | 0.22 | 0.35 |
| PL | 1 | 1 | 1 |  | 0.79 | 0.45 | 0.0229 | 0.0040 | 0.0229 |
| ILA | 1 | 0.17 | 1 | 0.79 |  | 1 | 1 | 0.66 | 1 |
| ORB | 1 | 1 | 1 | 0.45 | 1 |  | 1 | 0.16 | 0.34 |
| DP | 0.17 | 0.0094 | 1 | 0.0229 | 1 | 1 |  | 1 | 1 |
| TTd | 0.0187 | 0.0010 | 0.22 | 0.0040 | 0.66 | 0.16 | 1 |  | 1 |
| AON | 0.0549 | 0.0043 | 0.35 | 0.0229 | 1 | 0.34 | 1 | 1 |  |
|  | Motor | PFC | Olfactory |  |  |  |  |  |  |
| Motor |  | 0.78 | 6.80E-07 |  |  |  |  |  |  |
| PFC | 0.78 |  | 6.27E-09 |  |  |  |  |  |  |
| Olfactory | 6.80E-07 | 6.27E-09 |  |  |  |  |  |  |  |
| **Value-like cells (prop cue), Bonferroni corrected p-values for pairwise region comparisons (Figure 4-figure supplement 2, right)** | | | | | | | | | |
|  | ALM | ACA | FRP | PL | ILA | ORB | DP | TTd | AON |
| ALM |  | 1 | 1 | 1 | 1 | 1 | 0.57 | 1 | 0.42 |
| ACA | 1 |  | 1 | 1 | 1 | 1 | 1 | 1 | 1 |
| FRP | 1 | 1 |  | 1 | 1 | 1 | 1 | 1 | 1 |
| PL | 1 | 1 | 1 |  | 1 | 1 | 1 | 1 | 1 |
| ILA | 1 | 1 | 1 | 1 |  | 1 | 1 | 1 | 1 |
| ORB | 1 | 1 | 1 | 1 | 1 |  | 1 | 1 | 1 |
| DP | 0.57 | 1 | 1 | 1 | 1 | 1 |  | 1 | 1 |
| TTd | 1 | 1 | 1 | 1 | 1 | 1 | 1 |  | 1 |
| AON | 0.42 | 1 | 1 | 1 | 1 | 1 | 1 | 1 |  |
|  | Motor | PFC | Olfactory |  |  |  |  |  |  |
| Motor |  | 0.37 | 0.092 |  |  |  |  |  |  |
| PFC | 0.37 |  | 0.63 |  |  |  |  |  |  |
| Olfactory | 0.092 | 0.63 |  |  |  |  |  |  |  |
